# Embolism—Sudden Death

**Published:** 1864-10

**Authors:** 


*Embolism—Sudden Death.*

A woman, aged twenty-seven, was admitted a short time ago under M. Briquet, at the *Charite Hospital* of Paris. She was affected with considerable varicose dilatation of the saphena vein up to the groin, and improved considerably by rest and an appropriate treatment. The patient looked forward to a speedy discharge from the hospital, when one morning, whilst taking a cup of chocolate, she felt suddenly faint and died in twenty minutes. On a post-mortem examination, a clot was found in the pulmo-

---

nary artery, just at the origin of the division of the vessel. The clot was about five inches (?) in length and completely filled the vessel. It was reddish and hard, and contained coagulated fibrin. From its diameter it seemed to come from the iliac vein. Other portions of the vascular system were found quite normal.

M. Velpeau, recently read before the Academy of Sciences a highly interesting paper "*On Embolism*," taking for text a case of sudden death caused by a like vascular obturation which lately occurred in his wards at the *Charite*: A female, aged forty-six, in good health, was brought into hospital with a comminuted fracture of the right leg. The swelling being considerable, three weeks elapsed before the limb was set, and at the expiration of that period a dextrine bandage was applied. Two days after, whilst talking and laughing merrily with her neighbours in the ward, and without any warning, she shrieked out that she felt herself dying, and before the house-surgeon, who was summoned at once, arrived, she had expired. "It has been observed as an axiom," remarked M. Velpeau, "since the days of Bichat, that the causes of death are traceable to one of three organs—the brain, the heart, and the lung. Which, in this particular instance, was the organ at fault? Not the brain, for sudden death, as M. Flourens has proved, is very rare as emanating from the brain; nor the heart, for even after rupture of this organ, or of its great vessels, death is not immediate, and moreover, in such cases the face is pale, whilst here the features were livid and swollen. The source of death was, therefore, in the lung. This conclusion was verified by the autopsy: A clot was found to occupy the pulmonary artery, the ventricular orifice of which was thereby completely closed. The concretion formed in another part of the body, (in the iliac vein) had traversed the vena-cava, and so gained the heart and become impacted into the arterial channel of the lung, causing stoppage of its circulation and asphyxia. The remains of the original clot were still observable in the iliac vein, and the identity of texture and correspondence of measurements left no doubt as to the fact of the pulmonary clot having been detached from that in the iliac region. "An accident so common," added M. Velpeau, "and one so rapidly fatal, deserves the attention of the scientific community." Although migratory clots may and do occur in the arterial as well as in the venous system, and although in the former case the obliteration of a large trunk and consequent arrest of circulation may lead to the death of the part whose vascular supply is thus cut off, still the real gravity of embolism is confined to those instances in which the concretion exits in the channels leading directly to the right side of the heart and pulmonary vessels. The pulmonary circulation, as suggested by Dr. Ball in his thesis, contains three orders of vessels, each of which may become the seat of embolic concretions; we may find them, therefore, in arteries, capillaries, or veins. After having penetrated into the main artery of the lung, the migratory clot is either arrested by the primary, secondary, or testiary ramifications or else may pass on into the capillaries. The obstruction appears for the most part as a whitish mass, unconnected with the walls of the vessel in which it lies, and surrounded by the reddish coagulum deposited after its impaction. The character of this subsequent coagulum, its stratification, its extent, its puriform or fatty degeneration, constitute so many elements for speculation upon the date of the embolic migration. The passage of a clot, according to Dr. Ball, from one part of the venous system to the pulmonary ramifications, when not productive of immediate death, occasions inflammation of the obstructed vessel, with adhesion of the obstacle to the arterial walls, congestion of the lung, and other consequent pathological modifications, of which gangrene is one of the gravest. With regard to the constitutional causes which favour embolism, the author mentions—besides anæmia and cachexia, diphtheria; also albuminuria—a condition in which the fibrin of the blood being increased, the chances of spontaneous coagulation are materially augmented; and lastly and chiefly, the puerperal state. The principal symptoms of pulmonary embolism are the sudden appearance of dyspnœa, not to be accounted for by auscultation or percussion, coldness of the surface; and, as M. Velpeau remarks, livid discoloration of the face, together with perfect integrity of the intellectual functions. With regard to the therapeutical measures for the treatment of embolism, medical science is well nigh powerless; but for its prevention, and for the avoidance of all those conditions which are known to favour its production, much may be done; and to the employment of digitalis, Dr. Ball more especially directs attention as the means best calculated to avert the return of symptoms of embolism, should they already once have manifested themselves.

